# Measurement of 3-D Vibrational Motion by Dynamic Photogrammetry Using Least-Square Image Matching for Sub-Pixel Targeting to Improve Accuracy

**DOI:** 10.3390/s16030359

**Published:** 2016-03-11

**Authors:** Hyoseong Lee, Huinam Rhee, Jae Hong Oh, Jin Ho Park

**Affiliations:** 1 Department of Civil Engineering, Sunchon National University, Sunchon 57922, Korea; hslee@sunchon.ac.kr; 2 Department of Mechanical & Aerospace Engineering, Sunchon National University, Sunchon 57922, Korea; 3 Department of Civil Engineering, Chonnam National University, Gwangju 61186, Korea; ojh@chonnam.ac.kr; 4 Nuclear Technology Convergence Division, Korea Atomic Energy Research Institute, Daejeon 34057, Korea; pjh213@kaeri.re.kr

**Keywords:** displacement sensor, 3-D vibration, digital close-range photogrammetry, correlation matching, least square matching, sub-pixel targeting

## Abstract

This paper deals with an improved methodology to measure three-dimensional dynamic displacements of a structure by digital close-range photogrammetry. A series of stereo images of a vibrating structure installed with targets are taken at specified intervals by using two daily-use cameras. A new methodology is proposed to accurately trace the spatial displacement of each target in three-dimensional space. This method combines the correlation and the least-square image matching so that the sub-pixel targeting can be obtained to increase the measurement accuracy. Collinearity and space resection theory are used to determine the interior and exterior orientation parameters. To verify the proposed method, experiments have been performed to measure displacements of a cantilevered beam excited by an electrodynamic shaker, which is vibrating in a complex configuration with mixed bending and torsional motions simultaneously with multiple frequencies. The results by the present method showed good agreement with the measurement by two laser displacement sensors. The proposed methodology only requires inexpensive daily-use cameras, and can remotely detect the dynamic displacement of a structure vibrating in a complex three-dimensional defection shape up to sub-pixel accuracy. It has abundant potential applications to various fields, e.g., remote vibration monitoring of an inaccessible or dangerous facility.

## 1. Introduction

Measurement of structural vibration is a very important subject in various science and engineering fields. The most common sensors used to measure structural vibration may be different types of accelerometers, which require attaching them to the structure. In many cases this kind of contact-type sensor is inconvenient to use, and often the size and additional mass of the sensor may distort the vibrational characteristics of the structure. In some applications, such as monitoring vibration of a rotating shaft, proximity sensors are used to measure the displacement or velocity by non-contacting methods, however, they have a limited measurement distance and are difficult to use for general purposes. Recently, laser sensors are being widely used for detecting vibrational motion, but they are relatively expensive and have some limitation in the measurement distance and direction. It is noted that these methods do not provide a 3-D full-field measurement.

In some recent researches, a new non-contact method to measure the structural vibration has been proposed [1]. This method utilizes the digital close-range photogrammetry which was used for measuring the shapes using photo images. Photogrammetry is considered as the best technique for measuring the 3-D shape of turbine blades compared to optical triangulation and Moiré contour [2]. Photogrammetry has been used in analyzing aerial and satellite pictures, too [3,4,5]. Photogrammetry can be applied to the measurement of the dynamic deformation [4,6,7,8,9,10]. However, it is mostly focused on the field application of commercial high-speed camera systems without dealing with details about the measurement theory. Jeon *et al.* [11] applied the image processing to measure the vibration of a beam using a single camera, however, it was limited to 2-D measurement. Belen Ferrer *et al.* [12] developed a method to measure the vibration using an image processing to detect a sub-pixel movement of a structure. However, this method is limited to measure only the frequency from a 2-D image sequence, and cannot be applied to measure the 3-D dynamic displacement of a structure.

Lee and Rhee [1] have developed an efficient method to measure the full-field 3-D vibration in recent research. They introduced a theoretical basis of the digital photogrammetric method to measure the vibration using two inexpensive general purpose digital cameras without expensive high-speed cameras. Target matching was performed using the correlation coefficient. Then, the collinearity and space intersection were applied to trace the spatial position of each target. Finally, they performed an experiment to show that the theory can capture the dynamic 3-D displacements of a beam vibrating in a pure sinusoidal pattern with its fundamental natural frequency. In their work, however, the target matching was based on the pixel size of digital images. This means that the accuracy was limited to the size of one pixel, therefore, it depends on the resolution of the digital image and also the distance between the camera and the structure.

In this paper, we introduce a new methodology to improve the measurement accuracy by applying the least-square image matching for sub-pixel targeting. To determine the orientation parameters the space resection method is used rather than the bundle adjustment which was used in [1]. To verify the validity of the proposed method, a series of tests were performed to measure the dynamic displacements of a cantilever beam vibrating in mixed bending and torsional modes with multiple frequencies under the forced vibration condition.

## 2. Theory of 3-D Vibrational Displacement Measurement by Dynamic Photogrammetry Using Least Square Image Matching for Sub-Pixel Targeting

**Figure 1:**
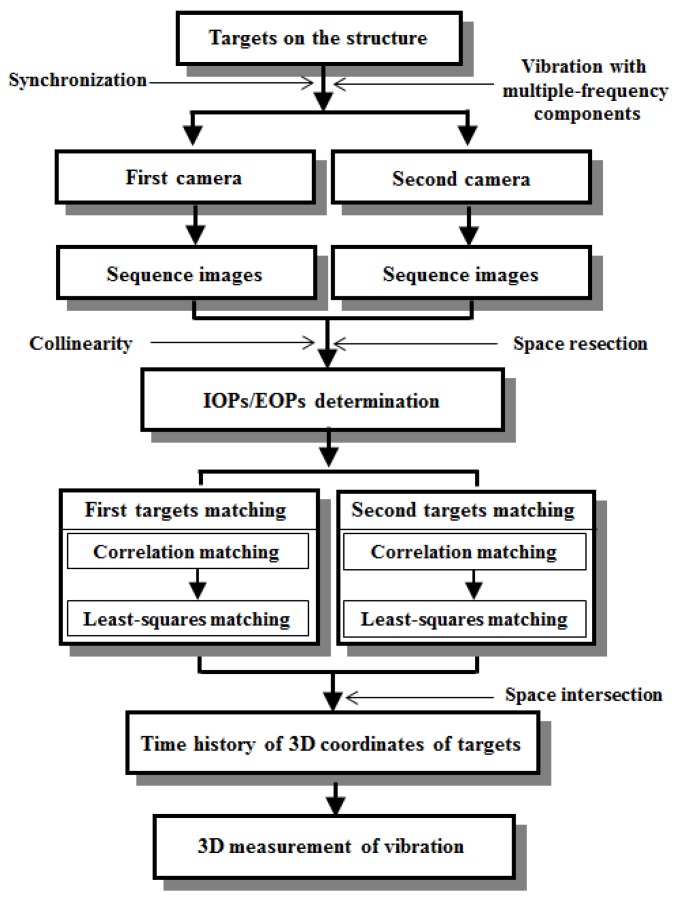
Proposed procedure for 3-D vibration measurement by stereo digital photogrammetry.

**Figure 2:**
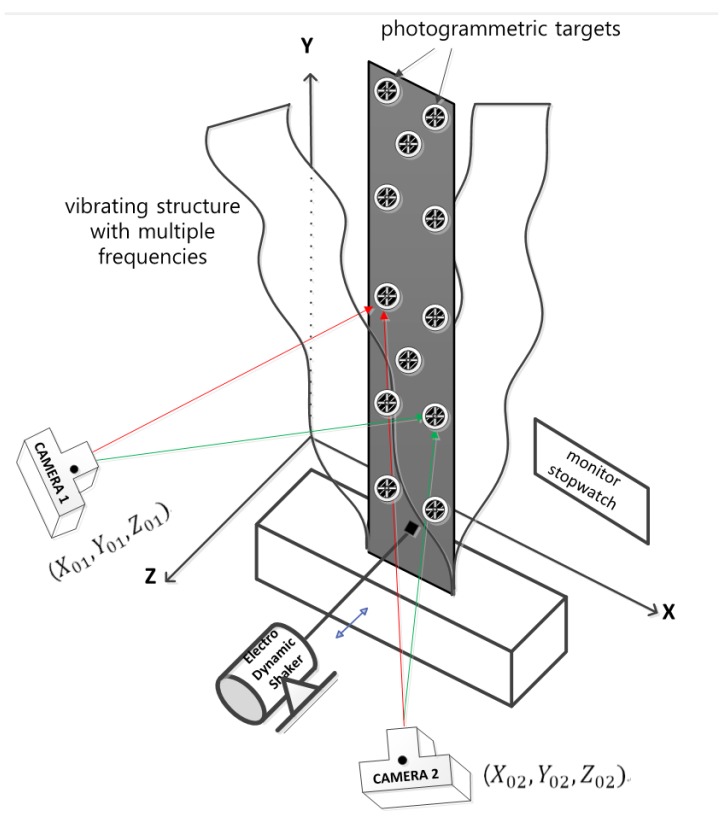
Schematic of the vibration measurement.

Figure 1 describes the overall procedure developed in this research for the measurement of the dynamic displacement of a vibrating object. In order to detect three-dimensional vibration, including both in-depth and out-of-plane motion by the dynamic photogrammetry, more than two cameras are required: in this paper, as a first step, stereo cameras are assumed for the development of the theory. Figure 2 shows the schematic of the vibration measurement in this study.

First of all, photogrammetric targets are installed at several fixed control points located near the vibrating structure. These targets are not shown in Figure 2 for simplicity. These are used as control points to determine IOPs (Interior Orientation Parameters) and EOPs (Exterior Orientation Parameters). IOPs consist of three parameters for each camera: focal length ($f_{i})$ and displacement of the principal point ($x_{0i}\;$ and $y_{0i}$) in the CCD image plane (i = 1 for left camera, i = 2 for right camera). EOPs include six parameters for each camera: the location (three spatial coordinates) of the lens center and the attitude (three rotation angles) of the lens in 3-D space.

As a next step, additional photogrammetric targets are attached to the vibrating structure, of which we want to measure the vibration, as shown in Figure 2. The dynamic movements of these targets are to be traced by the stereo photogrammetric technique in this study.

After installation of all photogrammetric targets, photo sequence of the vibrating structure is taken using two digital cameras, which may be either high-speed or cheap daily-use ones. The stereo images are synchronized using an appropriate electronics or a simple digital monitor stop-watch. In the experiment discussed later, we use the inexpensive daily-use cameras and a digital stop-watch as depicted in Figure 2 so that sophisticated expensive devices are not used in this study at all.

**Figure 3:**
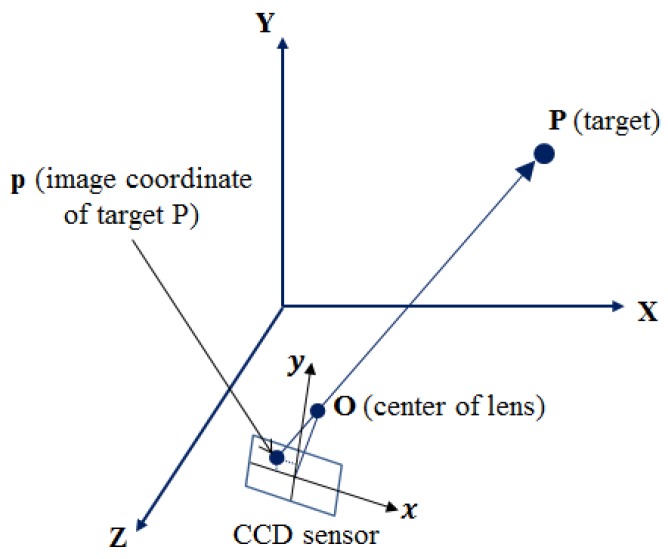
Collinearity condition.

In order to detect the dynamic motion by the dynamic photogrammetry, it is a prerequisite to determine IOPs and EOPs. In this research these parameters are computed by the space resection method using the collinearity condition in Equation (1), which refers to the linear alignment of the perspective center of the camera lens, the image points on CCD, and the points in the object space which coincide with the bundle of rays as shown in Figure 3 [13].

(1) $$\begin{array}{l} x_{ijk}-x_{oi}=-f_{i}\frac{r_{i,11}\left(X_{k}-X_{oij}\right)+r_{i,12}\left(Y_{k}-Y_{oij}\right)+r_{i,13}\left(Z_{k}-Z_{oij}\right)}{r_{i,31}\left(X_{k}-X_{oij}\right)+r_{i,32}\left(Y_{k}-Y_{oij}\right)+r_{i,33}\left(Z_{k}-Z_{oij}\right)}\\ y_{ijk}-y_{oi}=-f_{i}\frac{r_{i,21}\left(X_{k}-X_{oij}\right)+r_{i,22}\left(Y_{k}-Y_{oij}\right)+r_{i,23}\left(Z_{k}-Z_{oij}\right)}{r_{i,31}\left(X_{k}-X_{oij}\right)+r_{i,32}\left(Y_{k}-Y_{oij}\right)+r_{i,33}\left(Z_{k}-Z_{oij}\right)} \end{array}$$

where *i* = 1, 2 (1 = left camera, 2 = right camera), *j* = 1~α (α = number of photo images), *k* = 1~β (β = number of control points), $x_{ijk}$ and $y_{ijk}$ = image coordinates of targets, $x_{oi}$ and $y_{oi}$ = the coordinates of the principal point (image center), $X_{oij}$, $Y_{oij}$, and $Z_{oij}$ = spatial coordinates of the camera lens center, $X_{k}$, $Y_{k}$, and $Z_{k}$ = spatial coordinates of targets, *f_i_* = focal length. The information on the attitude of cameras are included in the rotational matrix components, $r_{i,11},r_{i,12}\cdots ,r_{i,33}$, which contain rotational angles $\left(\omega_{i},\;\phi_{i},\;\kappa_{i}\right)$ with respect to the *X*, *Y*, *Z* coordinates, respectively [1].

The 3-D space coordinates (*X*, *Y*, *Z*) and image coordinates (*x*, *y*) of the fixed control points can be determined by the total station and the image processing of the first set of stereo images (*j* = 1), respectively. Then, by substituting these coordinates into the collinearity in Equation (1), IOPs (*x_oi_*, *y_oi_*, and *f_i_*) and EOPs ($X_{oij}$, $Y_{oij}$, $Z_{oij}$, and $\omega_{i},\;\phi_{i},\;\kappa_{i}$) of left and right cameras can be determined. Therefore, the total number of orientation parameters is nine for each camera; therefore, at least nine fixed control points are required.

**Figure 4:**
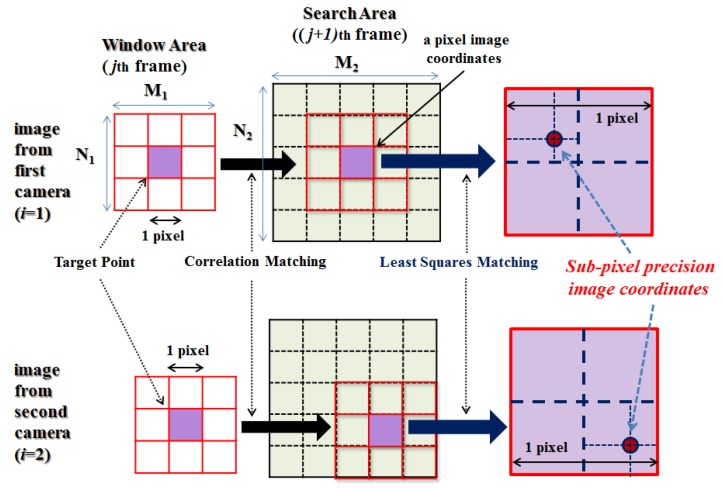
Proposed method using combined correlation and least square matching to obtain the sub-pixel accuracy.

Once the IOPs and EOPs are identified, the collinearity condition Equation (1) can be again utilized to compute the spatial coordinates $X_{k}$, $Y_{k}$, and $Z_{k}$ of targets attached to the vibrating structure if the corresponding image coordinates $x_{ijk}$ and $y_{ijk}$ are pre-determined. It is noted that now the subscript *k* in Equation (1) should represent the targets attached to the structure rather than the control points. The image coordinates of targets of the first of set of stereo image are obtained using an image processing technique. From the second set of images, the target-matching technique automatically traces the dynamic displacements of each target. In this study this procedure consists of two sub-steps as described in Figure 4. First, the correlation-matching is performed to roughly estimate the new location of targets with an accuracy of the unit pixel using the normalized correlation coefficient. Then, the least-squares-matching is applied to compute the new location with the sub-pixel precision in a finer manner.

In more detail, in the correlation-matching, the reference area contains a target at the center. Then the search area is constructed where the target is likely to locate after movement. The reference area is shifted by a pixel size step within the whole search area, and at every shift the correlation coefficient *Corr*(*m*, *n*) is determined using Equation (2). The probability of good matching is higher as *Corr*(*m*, *n*) approaches 1, therefore, the coordinates of targets are determined where *Corr*(*m*, *n*) has the maximum value within the search area:

(2) $$Corr(m,n)=\frac{\sum \sum \{S(x,y)-\bar{S}\}\{W(x,y)-\bar{W}\}}{{\left[\sum_{x=m}^{m+M_{1}-1}\sum_{y=n}^{n+N_{1}-1}{\left\{S(x,y)-\bar{S}\right\}}^{2}\sum_{x=1}^{M_{1}}\sum_{y=1}^{N_{1}}{\left\{W(x,y)-\bar{W}\right\}}^{2}\right]}^{1/2}}$$

where S(x,y) and W(x,y) are the pixel values in the search and reference areas, respectively. $M_{1}$ and $N_{1}$ are line and column pixel size in the reference area, respectively. The mean values in Equation (2) are computed as Equation (3):

(3) $$\bar{S}=\{\sum_{x=m}^{m+M_{1}-1}\sum_{y=n}^{n+N_{1}-1}S(x,y)\}/(M_{1}\times N_{1}),\bar{W}=\{\sum_{x=1}^{M_{1}}\sum_{y=1}^{N_{1}}W(x,y)\}/(M_{1}\times N_{1})$$

In Equations (2) and (3) the coordinates (x,y) are an integer pair of the multiples of unit pixel, so the resolution of the dynamic image coordinates is the unit pixel. In this paper a further step to more accurately determine the image coordinates is employed using the least-squares matching.

To define the discrepancy in the *W*(*x*, *y*) and *S*(*x*, *y*) between reference and search areas, every pixel value in the reference area is expressed as the corresponding radiometrically and geometrically transformed pixel values in the search area as follows [2]:

(4) $$\begin{array}{c} W_{k}\left(x,y\right)-e_{k}\left(x,y\right)=r_{0}+r_{1}S_{k}\left(x_{s},y_{s}\right)\\ x_{s}=a_{1}+a_{2}x+a_{3}y\\ y_{s}=b_{1}+b_{2}x+b_{3}y \end{array}$$

where, $k=1,\ldots ,M_{1}N_{1}.e_{k}\left(x,y\right)\;\text{is the noise component},$ $\text{and}\;(x_{s},y_{s})$ is the corresponding coordinate with position (*x*, *y*) in the reference image. $r_{0}$ and $r_{1}$ are radiometric-shift and -scale, respectively, for contrast and brightness (or equivalently offset and gain). In the present experimental study, which will be discussed in the following section, the effect of radiometric variation is not of concern, so these two parameters can be omitted but are included here for more general formulation for later use. $a_{1},a_{2},a_{3},b_{1},b_{2},\text{and}\;b_{3}$ are affine parameters for geometric transformation (a rotation, non-perpendicularity of the rotated two axes, two scale changes and two translation). Coordinates $x_{s}\;\text{and}\;y_{s}$ are not integer values any more; therefore, the corresponding pixel values are interpolated using the bilinear transformation.

Equation (4) should be linearized by Equation (5) to perform the least-squares technique (omitting the index *k* for simplicity).

(5) $$\begin{array}{c} W\left(x,y\right)-e\left(x,y\right)=S^{o}\left(x,y\right)+\frac{\partial S^{o}\left(x,y\right)}{\partial a_{1}}da_{1}+\frac{\partial S^{o}\left(x,y\right)}{\partial a_{2}}da_{2}+\frac{\partial S^{o}\left(x,y\right)}{\partial a_{3}}da_{3}+\\ \frac{\partial S^{o}\left(x,y\right)}{\partial b_{1}}db_{1}+\frac{\partial S^{o}\left(x,y\right)}{\partial b_{2}}db_{2}+\frac{\partial S^{o}\left(x,y\right)}{\partial b_{3}}db_{3}+r_{0}+r_{1}S^{o}\left(x,y\right) \end{array}$$

$S^{o}\left(x,y\right)$ is the approximation of the conjugate search patch. Since the areas are nearly aligned and are radiometrically similar, we can set initial parameter value to be $a_{1}^{0}=a_{3}^{0}=b_{1}^{0}=b_{2}^{0}=r_{0}^{0}=0$, $a_{2}^{0}=b_{3}^{0}=r_{1}^{0}=1$*.* Equation (5) can be expressed as follows:

(6) $$\begin{array}{c} \frac{\partial S^{o}\left(x,y\right)}{\partial a_{1}}=r_{1}^{0}\frac{\partial S^{o}\left(x,y\right)}{\partial x}\frac{\partial x}{\partial a_{1}}=\frac{\partial S^{o}\left(x,y\right)}{\partial x}\\ \frac{\partial S^{o}\left(x,y\right)}{\partial a_{2}}=r_{1}^{0}\frac{\partial S^{o}\left(x,y\right)}{\partial x}\frac{\partial x}{\partial a_{2}}=\frac{\partial S^{o}\left(x,y\right)}{\partial x}x\\ \frac{\partial S^{o}\left(x,y\right)}{\partial a_{3}}=r_{1}^{0}\frac{\partial S^{o}\left(x,y\right)}{\partial x}\frac{\partial x}{\partial a_{3}}=\frac{\partial S^{o}\left(x,y\right)}{\partial x}y\\ \frac{\partial S^{o}\left(x,y\right)}{\partial b_{1}}=r_{1}^{0}\frac{\partial S^{o}\left(x,y\right)}{\partial y}\frac{\partial y}{\partial b_{1}}=\frac{\partial S^{o}\left(x,y\right)}{\partial y}\\ \frac{\partial S^{o}\left(x,y\right)}{\partial b_{2}}=r_{1}^{0}\frac{\partial S^{o}\left(x,y\right)}{\partial y}\frac{\partial y}{\partial b_{2}}=\frac{\partial S^{o}\left(x,y\right)}{\partial y}x\\ \frac{\partial S^{o}\left(x,y\right)}{\partial b_{3}}=r_{1}^{0}\frac{\partial S^{o}\left(x,y\right)}{\partial y}\frac{\partial y}{\partial b_{3}}=\frac{\partial S^{o}\left(x,y\right)}{\partial y}y\\ \frac{\partial S^{o}\left(x,y\right)}{\partial r_{0}}=1,\frac{\partial S^{o}\left(x,y\right)}{\partial r_{1}}=S^{o}\left(x,y\right) \end{array}$$

where, $\frac{\partial S^{o}\left(x,y\right)}{\partial x}\approx \frac{S\left(x+1,y\right)-S\left(x-1,y\right)}{2},\frac{\partial S^{o}\left(x,y\right)}{\partial y}\approx \frac{S\left(x,y+1\right)-S\left(x,y-1\right)}{2}$.

If the transformation parameters are written as the vector of unknowns ***X***, the partial derivatives as the design matrix ***A*** and the pixel value differences between the reference and search images as the vector of observations ***L***, then linearized correction equations are given as follows [2]:

(7) $$-e_{\left(k,1\right)}=A_{\left(k,l\right)}X_{\left(l,1\right)}-L_{\left(k,1\right)}$$

where, $X^{T}=\left[da_{1},\;da_{2},\;da_{3},\;db_{1},\;db_{2},\;db_{3},\;dr_{0},\;dr_{1}\right],$ *k* = $M_{1}N_{1}$, and *l* = number of unknown parameters (8). Equation (7) can be expressed as the least-squares form in order to compute parameters $da_{1},da_{2},da_{3},db_{1},db_{2},db_{3},dr_{0},dr_{1}$ using Equation (8):

(8) $$X={\left(A^{T}A\right)}^{-1}\left(A^{T}L\right)$$

The adjustment equations must be solved iteratively. In every iteration the unknowns are updated using the result from Equation (8) such as $a_{1}^{1}=a_{1}^{0}+da_{1},a_{2}^{1}=a_{2}^{0}+da_{2},\ldots$. This leads to new pixel value differences between the reference and search images until the least-squares sum of the corrections is less than a predefined threshold (nearly zero). Finally, more accurate image coordinates after the dynamic deformation can be determined as depicted in Figure 4.

**Figure 5:**
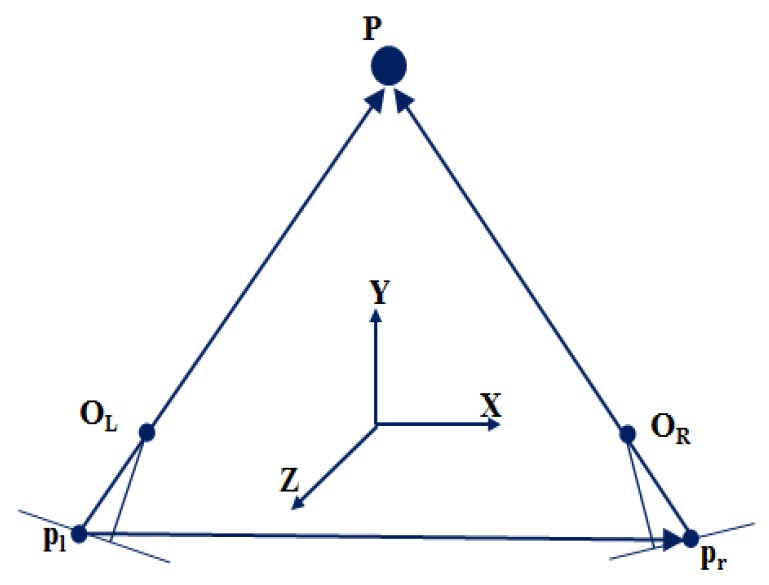
3-D positioning by space intersection of stereo pairs of digital images.

As a next step, 3-D coordinates of targets are obtained using the space intersection, in which the interior and exterior orientation parameters and the image coordinates of the same point in a set of stereo images are used. That is, once identical points are found in each stereo image by using the correlation and least-squares matching technique as explained above, two straight lines passing through p_l_-O_L_ and p_r_-O_R_ must intersect at a point P in Figure 5 [13].

The collinearity equation, Equation (1), can be rewritten for an arbitrary point as Equation (10):

(9) $$\begin{array}{c} A_{1}X+B_{1}Y+C_{1}Z+D_{1}=0\\ E_{1}X+F_{1}Y+G_{1}Z+H_{1}=0\\ A_{2}X+B_{2}Y+C_{2}Z+D_{2}=0\\ E_{2}X+F_{2}Y+G_{2}Z+H_{2}=0 \end{array}$$

where, X,Y,Z = 3-D spatial coordinates of a target, sub-index = camera number, and coefficients are as follows:

(10) $$\begin{array}{l} A_{1}=\left(x-x_{o1}\right)r_{1,31}+f_{1}r_{1,11};B_{1}=\left(x-x_{o1}\right)r_{1,32}+f_{1}r_{1,12};C_{1}=\left(x-x_{o1}\right)r_{1,33}+f_{1}r_{1,13};\\ D_{1}=-\left(x-x_{o1}\right)r_{1,31}X_{o1}-f_{1}r_{1,11}\;X_{o1}-\left(x-x_{o1}\right)r_{1,32}Y_{o1}-f_{1}r_{1,12}Y_{o1}\;-\left(x-x_{o1}\right)r_{1,33}Z_{o1}-f_{1}r_{1,13}Z_{o1}\\ E_{1}=\left(y-y_{o1}\right)r_{1,31}+f_{1}r_{1,21};F_{1}=\left(y-y_{o1}\right)r_{1,32}+f_{1}r_{1,22};G_{1}=\left(y-y_{o1}\right)r_{1,33}+f_{1}r_{1,23};\\ H_{1}=-\left(y-y_{o1}\right)r_{1,31}X_{o1}-f_{1}r_{1,21}\;X_{o1}-\left(y-y_{o1}\right)r_{1,32}Y_{o1}-f_{1}r_{1,22}Y_{o1}-\left(y-y_{o1}\right)r_{1,33}Z_{o1}-f_{1}r_{1,23}Z_{o1};\\ A_{2}=\left(x-x_{o2}\right)r_{2,31}+f_{2}r_{2,11};B_{2}=\left(x-x_{o2}\right)r_{2,32}+f_{2}r_{2,12};C_{2}=\left(x-x_{o2}\right)r_{2,33}+f_{2}r_{2,13};\\ D_{2}=-\left(x-x_{o2}\right)r_{2,31}X_{o2}-f_{2}r_{2,11}\;X_{o2}-\left(x-x_{o2}\right)r_{2,32}Y_{o2}-f_{2}r_{2,12}Y_{o2}-\left(x-x_{o2}\right)r_{2,33}Z_{o2}-f_{2}r_{2,13}Z_{o2};\\ E_{2}=\left(y-y_{o2}\right)r_{2,31}+f_{2}r_{2,21};F_{2}=\left(y-y_{o2}\right)r_{2,32}+f_{2}r_{2,22};G_{2}=\left(y-y_{o2}\right)r_{2,33}+f_{2}r_{2,23};\\ E_{2}=\left(y-y_{o2}\right)r_{2,31}+f_{2}r_{2,21};F_{2}=\left(y-y_{o2}\right)r_{2,32}+f_{2}r_{2,22};G_{2}=\left(y-y_{o2}\right)r_{2,33}+f_{2}r_{2,23};\\ H_{2}=-\left(y-y_{o2}\right)r_{2,31}X_{o2}-f_{2}r_{2,21}\;X_{o2}-\left(y-y_{o2}\right)r_{2,32}Y_{o2}-f_{2}r_{2,22}Y_{o2}-\left(y-y_{o2}\right)r_{2,33}Z_{o2}-f_{2}r_{2,23}Z_{o2} \end{array}$$

where some detailed sub-indexes are omitted from Equation (1) for simplicity.

Equation (10) can be arranged in a matrix form as Equation (11), and then 3-D spatial coordinates can be calculated by Equation (12) using the least-squares approach:

(11) $$\left[\begin{array}{c} \begin{array}{c} A_{1}\;B_{1}\;C_{1}\\ E_{1}\;F_{1}\;G_{1} \end{array}\\ \begin{array}{c} A_{2}\;B_{2}\;C_{2}\\ E_{2}\;F_{2}\;G_{2} \end{array} \end{array}\right]\left[\begin{array}{c} X\\ Y\\ Z \end{array}\right]=\left[\begin{array}{c} \begin{array}{c} -D_{1}\\ -H_{1} \end{array}\\ \begin{array}{c} -D_{2}\\ -H_{2} \end{array} \end{array}\right]$$

(12) $$\begin{array}{c} P\;T\;Q\\ T={\left(P^{T}\cdot P\right)}^{-1}P^{T}\cdot Q \end{array}$$

Thus, the 3-D dynamic displacement of vibrating structures can be measured with sub-pixel accuracy.

This methodology enables the full-field measurement of the vibrating structure because there is no limit in the number of targets. The targets are just a piece of papers or pen-marks, therefore, there is no mass-loading effects compared to the conventional sensors such as accelerometers. Moreover, when unique features on its surface and shape such as corners are utilized, even the targets may not be necessary. In the following section, an experiment is successfully performed to verify the proposed method to measure the 3-D vibration.

## 3. Experiment

**Figure 6:**
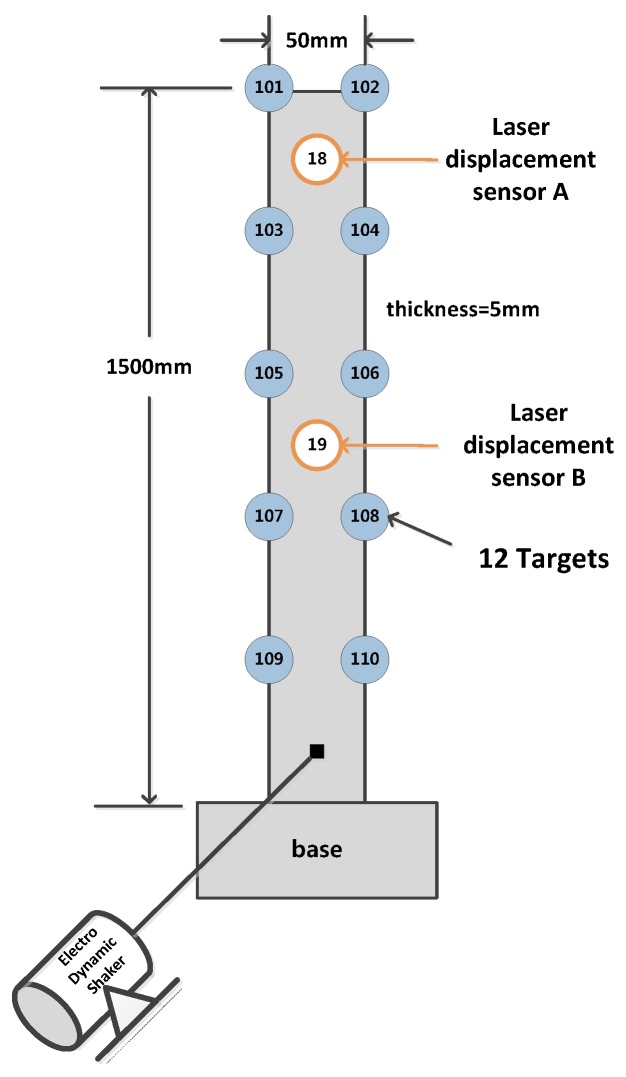
Cantilevered beam with multiple targets under forced excitation.

A series of experiments were performed to verify the proposed methodology for the 3-D vibration measurement using two cameras. A cantilever composite beam was fixed at its lower end as shown in Figure 6. An electrodynamic shaker was installed at its bottom part to excite the cantilever. The beam has 12 targets, and additional targets were attached at 16 control points near the cantilever beam. Two laser displacement sensors (Keyence LKG-5000), which are operated by optical triangulation, were installed at two locations, on the back side of targets 18 and 19, to verify the measurement accuracy of the proposed method. The photogrammetric system uses two cameras to take sequential photo images and a monitor stopwatch for the temporal synchronization of the stereo images. The camera is an inexpensive daily use camera, CASIO EX-FH20, which can take 40 fps. The resolution was 3072 pixels × 2304 pixels.

**Figure 7:**
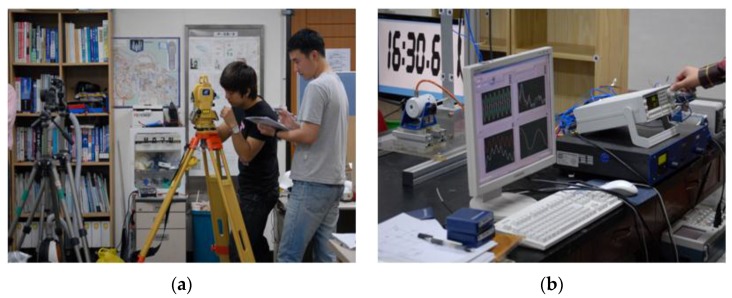
Reference points survey using the total-station and monitoring of forced excitation frequency. (**a**) Total-station; (**b**) Monitoring of the dynamic displacement.

The spatial coordinates of control points were determined by the total station, which is a distance measurement device used for the 3-D land survey, as shown in Figure 7.

**Figure 8:**
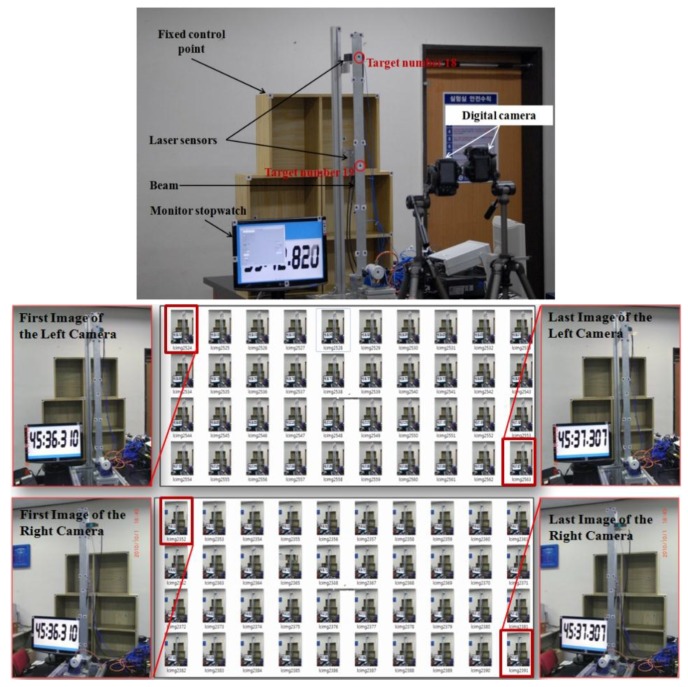
Measurement practice example (from **top** to **bottom**: test set-up, **left** and **right** camera images, respectively).

Forty sequential photographs which contain images of the vibrating beam, monitor stop-watch, and fixed control points were taken using two cameras, as shown in Figure 8. In a previous experience [1] the monitor stop-watch was proved to be reliable enough to synchronize stereo images without many trials. Of course, an electronic synchronization would be more accurate, but this research focuses on the improvement by adding the least square matching procedure for sub-pixel targeting while other conditions are kept as same as possible. Nine of the 16 control points were used for the determination of IOPs and EOPs. The other seven points were used later as checkpoints to verify the accuracy of the determined IOPs and EOPs.

The IOPs and EOPs of the two cameras in Table 1 were computed by the space resection method using the 3-D location of the control points and their image coordinates in the first stereo photo [14]. In order to verify the accuracy of the computed parameters, the 3-D coordinates of the seven checkpoints were computed by using Equation (12) and then the results were compared with the measurements by the total station. They showed accurate enough consistency within 0.1 mm root mean square error; therefore, it was decided that IOPs and EOPs could be a good basis to measure the vibration.

**Figure 9:**
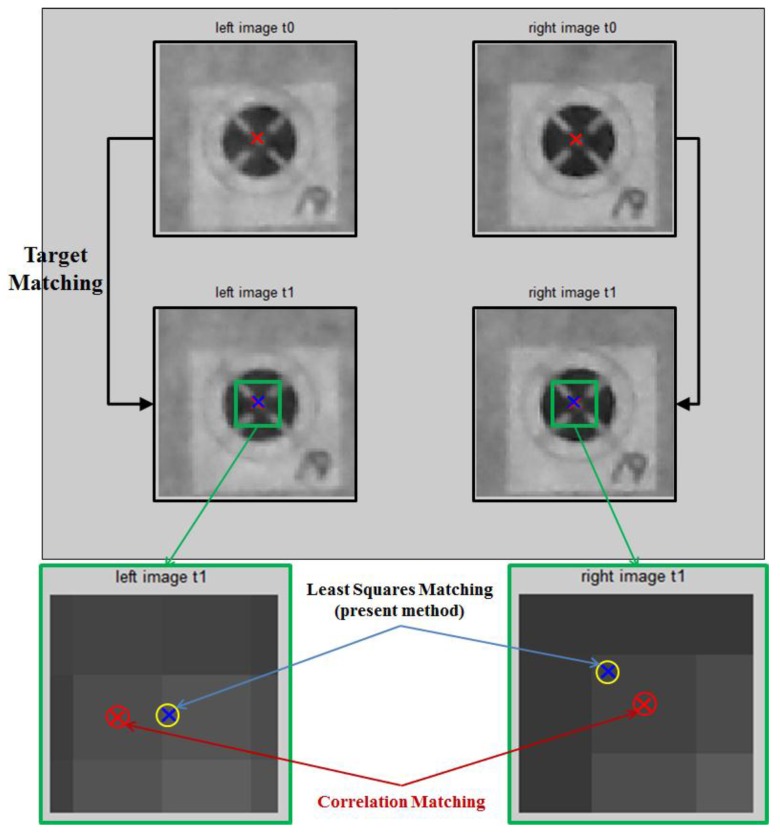
Comparison of correlation matching and least-squares matching.

Next, the image coordinates of targets installed at the vibrating structure were determined by applying the proposed method, that is, the correlation matching (Equation (2)) followed by the least-squares matching (Equation (8)) as depicted in Figure 4. Figure 9 shows an example which explains that the least-squares matching refines the location to the sub-pixel size accuracy. Then, as a final step, the 3-D spatial coordinates of targets are computed by Equation (12).

**Figure 10:**
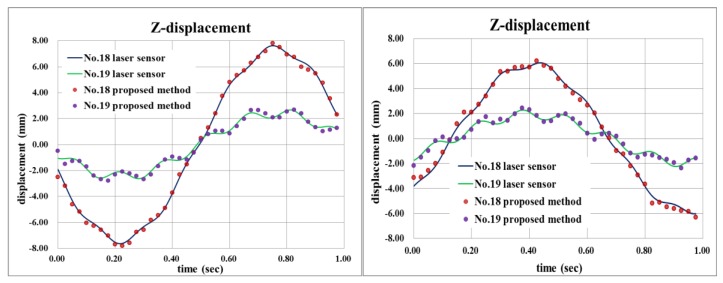
Comparison of out-of-plane vibrational displacements measured by the proposed method and laser sensors at target 18 and 19 positions (**left** and **right** correspond to different time interval).

**Figure 11:**
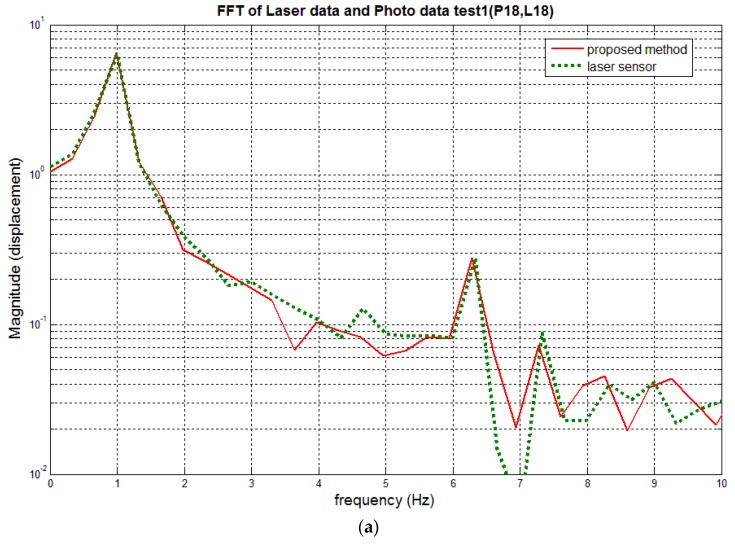

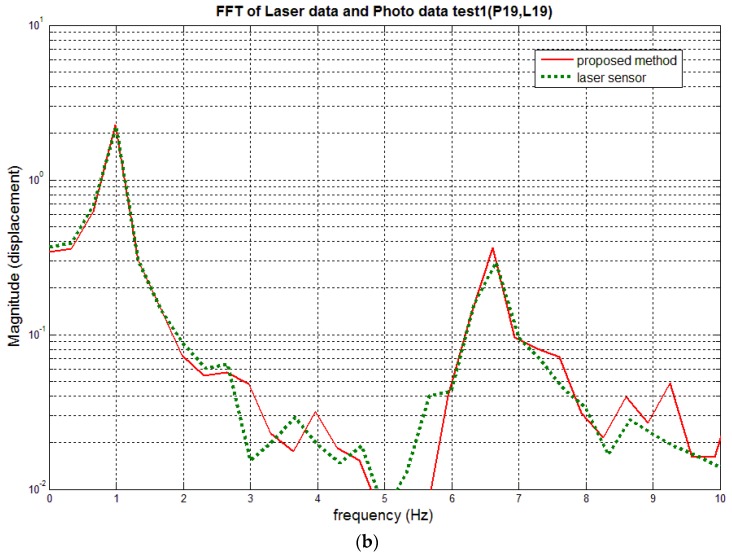
Comparison of out-of-plane displacement spectra measured by the proposed method and laser sensors at target 18 and 19 positions (for left curves of Figure 10). (**a**) Target 18 position; and (**b**) Target 19 position.

Figure 10 compares the out-of-plane dynamic displacements of the beam at targets 18 and 19 positions (see Figure 6) which are obtained using the proposed method with the laser displacement sensor measurement. The result clearly reveals that the dynamic 3-D displacement can be very accurately measured by the proposed method even though the structure vibrates in enough of a complex shape with multiple frequencies. The small discrepancy could be further reduced easily if a better-quality camera, e.g., a camera with lower lens distortion and electronic synchronization function, is used. Figure 11 compares their spectrum, and shows good consistency; therefore, the accuracy of the proposed method is again proved.

It is noted that the root mean square errors of the measurement by the proposed method relative to the laser sensor are 0.20 mm and 0.17 mm for targets 18 and 19, respectively. Maximum displacement at Target 18 is bigger (~8 mm) than that at target 19 (~2 mm), therefore, it is reasonable enough that the displacement at Target 18 has a larger root mean square error, although some other factors, such as different wave patterns at the two locations, may have influences on the measurement accuracy, too. In the previous study [1] which used solely correlation matching without least square matching, the root mean square error was 0.21 mm ([1] p. 68), which looks comparable to the present results. However, in the previous study, the maximum displacement was only 2 mm due to the shorter length of the cantilever beam and weaker excitation by the piezoelectric patch. Thus, the present results are actually more accurate considering the larger displacement and complex vibration pattern. The spectrum in Figure 11 also shows better accuracy compared to the spectrum in the reference [1] (Figure 9). In fact, Figure 9 clearly shows the effectiveness of the application of the least square matching. The locations of red and blue marks in Figure 9 may be quite different in some case, but may be very close in some other case: it would be almost random as the structure is vibrating. Therefore, it is obvious that the proposed method utilizing the least square matching will generally have much better accuracy while even in a worst case it will still have a slightly better accuracy compared to the correlation matching only.

**Figure 12:**
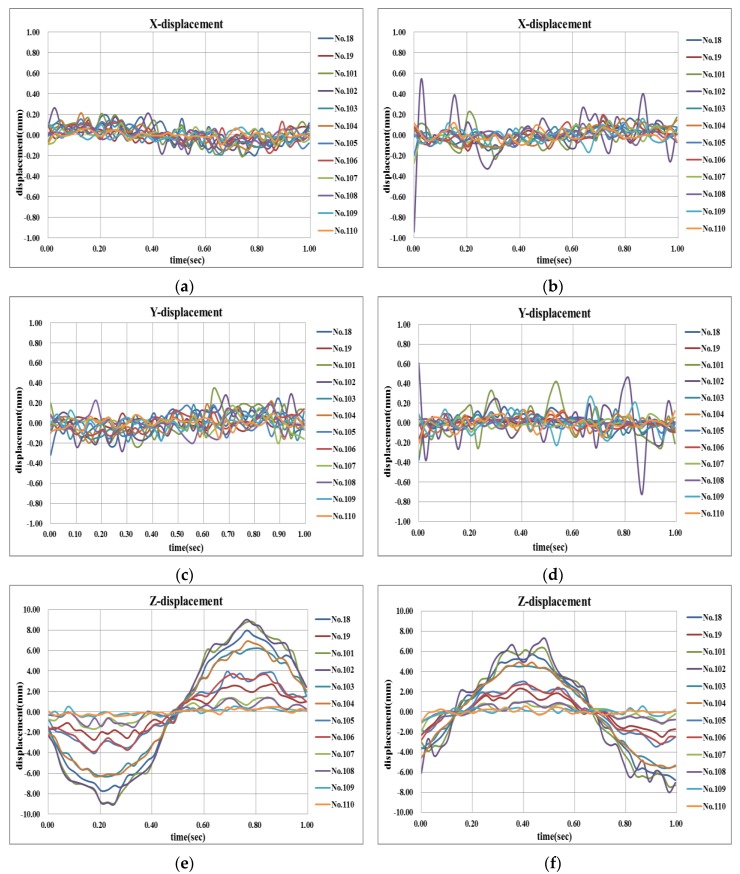
Examples of three-directional displacements measured by the proposed method (**left** and **right** columns correspond to different time interval). (**a**) x-displacement, time interval I; (**b**) x-displacement, time interval II; (**c**) y-displacement, time interval I; (**d**) x-displacement, time interval II; (**e**) z-displacement, time interval I; (**f**) x-displacement, time interval II.

**Figure 13:**
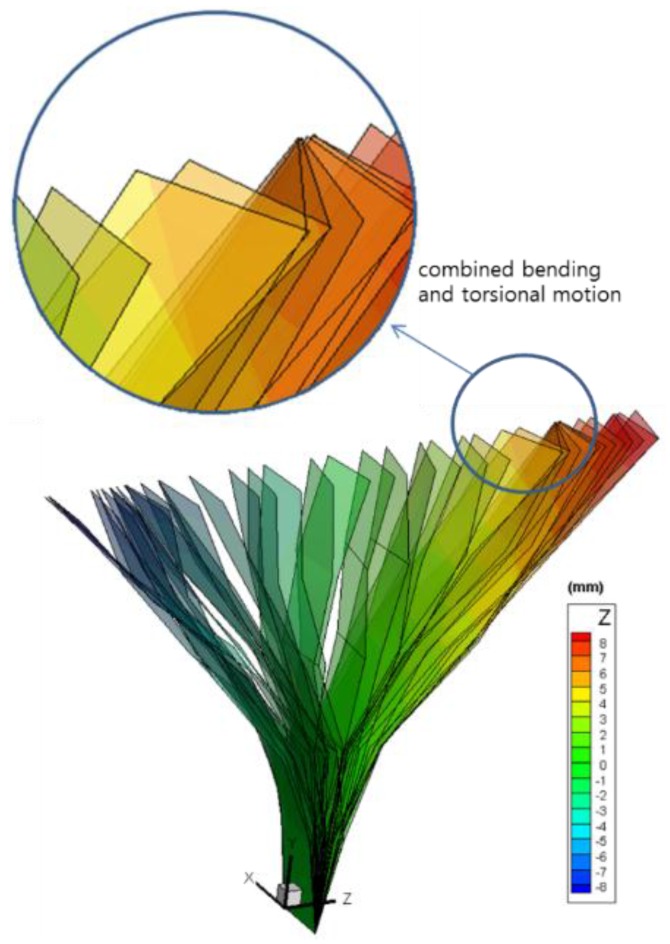
Consecutive vibration patterns during one second measured by the proposed method (different colors represent out-of-plane displacements).

Figure 12 shows the 3-D displacement time-histories at 12 target positions. Using Figure 12, the 3-D dynamic deformation shape of a vibrating structure, which is often referred to as an operational deflection shape (ODS), is directly obtained as shown in Figure 13. The beam in this experiment undergoes combined bending and torsional modes. It is revealed that even the subtle torsional motion is well captured by the proposed method. Figure 13 can also be easily displayed as a movie file so that one can visually understand the complex operational vibration characteristics in detail. It would be useful for the analysis or monitoring of vibrating structures in various fields, including non-accessible hazardous facility, automobile panels, *etc*.

If other type of sensors such as accelerometers or laser sensors are used to obtain the deformation shape in Figure 13, 36 unidirectional sensors or 12 three-axes sensors will be needed, which would be impossible or, at least, impractical. Moreover, contact-type sensors such as accelerometers would cause severe mass loading effects, so the original structural vibration characteristics are distorted. It is noted that even a highly-sophisticated laser scanning device cannot measure the 3-D operational deflection shape in Figure 13.

## 4. Conclusions

An improved three-dimensional dynamic displacement measurement method using close-range digital photogrammetry was proposed in this paper. The proposed method combines the correlation matching method and the least-squares matching technique, so that the measurement accuracy significantly increases compared to the correlation matching-only method in the previous study by the authors. This method can be easily applied using only cheap daily-use cameras to detect 3-D vibration information without expensive high-speed cameras. A series of experiments were successfully performed to verify the proposed method by measuring the displacement of a cantilever beam vibrating at multiple frequencies in combined bending and torsional modes using two cameras which take 40 frames per second. The accuracy has been verified by comparing the measurement results to the laser sensor measurement in time and frequency domains.

The proposed method has great advantages because it can perform the full-field measurement of three-dimensional dynamic displacements of a vibrating structure by non-contact methods. It does not necessarily require highly-sophisticated devices such as high-speed cameras. The proposed method can be applied to the vibration monitoring with improved accuracy in various industrial situations as well as in a laboratory condition. Remote monitoring of the vibration of bridges or buildings or non-accessible nuclear facilities may be good examples of the application.

## Figures and Tables

**Table 1:** IOPs and EOPs of the two cameras.

	f (mm)	$x_{0}$ (mm)	$y_{0}$ (mm)	$X_{0}$ (mm)	$Y_{0}$ (mm)	$Z_{0}$ (mm)	ω (rad)	ϕ (rad)	κ (rad)
Left Camera	6.38	0.07	0.10	1727.5	2410.3	4131.7	−0.0225	−0.1560	−0.0141
Right Camera	6.53	−0.11	−0.04	2505.1	2427.2	4125.1	−0.0118	0.2820	0.0116

## References

[1] Lee H., Rhee H. (2013). 3-D measurement of structural vibration using digital close-range photogrammetry. Sens. Actuators A Phys..

[2] Clarke T.A., Robson S., Chen J. A comparison of three techniques for the 3-D measurement of turbine blades. Proceedings of the ISMTII.

[3] Dold J. (1998). The role of a digital intelligent camera in automating industrial photogrammetry. Photogramm. Rec..

[4] Helfrick M.N., Niezrecki C., Avitabile P., Schmidt T. 3D Digital Image Correlation Methods for Full-Field Vibration Measurement. Proceedings of the Twenty Sixth International Modal Analysis Conference.

[5] Luhmann T., Robson S., Kyle S., Harley I. (2006). Close Range Photogrammetry Principles, Methods and Applications.

[6] Schmidt T., Tyson J. Performance Verification of 3D Image Correlation Using Digital High-Speed Cameras. Proceedings of the SEM Annual Conference & Exposition on Experimental and Applied Mechanics.

[7] Ghaem-Maghami E., Desabrais J., Johari H. Measurement of the Geometry of a Parachute Canopy Using Image Correlation Photogrammetry. Proceedings of the 19th AIAA Aerodynamic Decelerator Systems Technology Conference and Seminar.

[8] Helfrick M.N., Niezrecki C., Avitabile P., Schmidt T. (2011). 3D digital image correlation methods for full-field vibration measurement. Mech. Syst. Signal Process..

[9] Avitabile P., Niezrecki C., Helfrick M., Warren C., Pingle P. (2010). Noncontact Measurement Techniques for Model Correlation. Sound Vib..

[10] Ozbek M., Rixen D.J., Erne O., Sanow G. (2010). Feasibility of monitoring large wind turbines using photogrammetry. Energy.

[11] Jeon H.S., Choi Y.C., Park J.H., Park J.W. (2010). Multi-point measurement of structural vibration using pattern recognition from camera image. Nucl. Eng. Technol..

[12] Ferrer B., Espinosa J., Roig A.B., Perez J., Acevedo P., Mas D. (2014). Low cost subpixel method for vibration measurement. Proceedings of the 11th International Vibration Measurement by Laser and Noncontact Techniques AIVELA.

[13] Moffitt F.H., Mikhail E.M. (1980). Photogrammetry.

[14] Mikhail E.M., Bethel J.S., McGlone J.C. (2001). Introduction to Modern Photogrammetry.

